# Antihypertensive therapy, new-onset diabetes, and cardiovascular disease

**DOI:** 10.1111/j.1742-1241.2009.02009.x

**Published:** 2009-04

**Authors:** J N Basile

**Affiliations:** Primary Care Service Line, Ralph H. Johnson VA Medical Center and Department of Medicine, Medical University of South CarolinaCharleston, SC, USA

## Abstract

Type 2 diabetes mellitus is a worldwide epidemic with considerable health and economic consequences. Diabetes is an important risk factor for cardiovascular disease, which is the leading cause of death in diabetic patients, and decreasing the incidence of diabetes may potentially reduce the burden of cardiovascular disease. This article discusses the clinical trial evidence for modalities associated with a reduction in the risk of new-onset diabetes, with a focus on the role of antihypertensive agents that block the renin–angiotensin system. Lifestyle interventions and the use of antidiabetic, anti-obesity, and lipid-lowering drugs are also reviewed. An unresolved question is whether decreasing the incidence of new-onset diabetes with non-pharmacologic or pharmacologic intervention will also lower the risk of cardiovascular disease. A large ongoing study is investigating whether the treatment with an oral antidiabetic drug or an angiotensin-receptor blocker will reduce the incidence of new-onset diabetes and cardiovascular disease in patients at high risk for developing diabetes.

**Review Criteria**

The information used to prepare this manuscript was gathered by reviewing guidelines for treatment of prediabetes and from a PubMed search using the following keywords: ‘antihypertensive therapy’, ‘cardiovascular disease’, ‘impaired fasting glucose’, ‘impaired glucose tolerance’, ‘renin–angiotensin system’ and ‘type 2 diabetes mellitus’. The major randomised, controlled clinical trials evaluating the capacity of lifestyle interventions, antidiabetic drugs, lipid-lowering drugs, and antihypertensive drugs on the delay of new-onset diabetes and cardiovascular disease are reviewed herein.

**Message for the Clinic**

Evidence suggests that lifestyle modifications aimed at weight reduction and increased physical activity and antidiabetic pharmacologic interventions reduce the risk of new-onset diabetes. Although there is ample evidence that antihypertensive therapy with RAS inhibitors is associated with a reduced risk of new-onset diabetes compared with other classes of antihypertensive drugs, the prognostic significance of this differential effect remains controversial. For now, lifestyle measures and the reduction of global cardiovascular disease risk may be a more effective strategy to improve vascular health and limit insulin resistance in patients with hypertension than restricting the use of any particular antihypertensive agent.

The prevalence of type 2 diabetes mellitus is increasing worldwide. The number of people living with diabetes is expected to more than double from 171 million in 2000 to 366 million by 2030 (1). In the United States, an estimated 9.3% of adults aged 20 years or older have diabetes (6.5% diagnosed and 2.8% undiagnosed), a significant increase (p = 0.0002) from the 5.1% prevalence of diagnosed diabetes in the previous decade. Increases have been seen in all age groups, both sexes and all race/ethnic groups (2).

Risk factors for the development of diabetes include overweight or obesity, physical inactivity, hypertension, dyslipidaemia, family history, impaired glucose tolerance and impaired fasting glucose. Type 2 diabetes is an important risk factor for cardiovascular disease (3) and is regarded as a coronary heart disease ‘risk equivalent’ by the Adult Treatment Panel III of the National Cholesterol Education Program (4). Individuals with type 2 diabetes have a two- to threefold higher risk of cardiovascular disease than their non-diabetic counterparts (5), and, in the United States, the majority (65%) of deaths in people with diabetes are due to heart disease and stroke (6). Diabetes is also associated with a significant increase in risk of concomitant hypertension and dyslipidaemia (7).

The higher incidence of cardiovascular disease in patients with type 2 diabetes can be explained in part by the increased prevalence of comorbid risk factors (8,9). However, even after correction for these factors, diabetes confers a 1.5- to 4.5-fold increase in risk of myocardial infarction in women, a 1.5- to twofold increase in risk of myocardial infarction in men, and a 1.5- to twofold increase in risk of death in both sexes (10).

Chronic hyperglycaemia is the defining characteristic of diabetes and the target of antidiabetic therapy but the benefits of reducing elevated glucose values and the specific target that should be achieved remain uncertain. While maintenance of glycaemic control has been shown to reduce the risk of microvascular complications, including retinopathy, nephropathy and neuropathy (11,12), studies of the effect of glycaemic control on the risk of macrovascular complications have produced conflicting results. A meta-analysis of prospective cohort studies that assessed the association between glycosylated haemoglobin (A1C) levels and macrovascular disease in patients with diabetes observed that, in patients with type 2 diabetes, each one-percentage point increase in A1C is associated with an 18% increase in risk of cardiovascular disease (13). In the United Kingdom Prospective Diabetes Study, intensive glycaemic control to an average A1C of 7% in patients with type 2 diabetes over a 10-year follow-up period reduced the risk of microvascular, but not macrovascular, complications compared with an average A1C of 7.9% (12). In the 6.5-year Diabetes Control and Complications Trial (DCCT), intensive treatment compared with conventional treatment in patients with type 1 diabetes was associated with 76% and 54% reductions in the risk of development and progression of retinopathy, respectively (p< 0.001 for both), but no reduction was noted in macrovascular disease (11). It was, however, in the Epidemiology of Diabetes Interventions and Complications Study, an 11-year followup to DCCT, where intensive treatment reduced the risk of any cardiovascular disease event by 42% (p= 0.02) and the risk of non-fatal myocardial infarction, stroke, or death from cardiovascular disease by 57% (p= 0.02) (14).

Two recently published landmark trials – the Action in Diabetes and Vascular Disease: Pretaraz and Diamicron Modified Release Controlled Evaluation (ADVANCE) (15) and the Action to Control Cardiovascular Risk in Diabetes (ACCORD) (16) – failed to demonstrate that intensive glycaemic control reduces cardiovascular disease risk in those with long-standing type 2 diabetes. In both the 5-year ADVANCE trial and the planned 4- to 8-year ACCORD trial, intensive glucose control (defined as a target A1C level < 6.5% in ADVANCE and < 6.0% in ACCORD) had no significant effects on the incidence of cardiovascular disease events compared with standard glucose control. Moreover, in ACCORD, a significant increase in all-cause mortality in the intensive treatment group led to premature discontinuation of that arm of the trial at 3.5 years. Taken together, the results of ADVANCE and ACCORD suggest that 7% remains an appropriate A1C target in those with long-standing type 2 diabetes (17,18). While the results of the previous trials (including ADVANCE) confirm the role of more aggressive glycaemic control in reducing the risk of microvascular complications, the lack of effect on cardiovascular disease events may have important implications for our understanding of the pathogenesis and reversibility of macrovascular complications of diabetes. Taken together, these results suggest the need for earlier intervention in those with type 2 diabetes as well as the need to address non-glycaemic comorbid risk factors, such as hypertension and dyslipidaemia (18).

## New-onset diabetes and cardiovascular disease

The current criteria for diagnosis of diabetes are a fasting plasma glucose level ≥ 126 mg/dl, symptoms of hyperglycaemia, and a casual plasma glucose level ≥ 200 mg/dl, or a 2-h postchallenge plasma glucose level ≥ 200 mg/dl during an oral glucose tolerance test; fasting plasma glucose < 100 mg/dl and 2-h plasma glucose < 140 mg/dl are considered ‘normal’ (19). The intermediate hyperglycaemic state that does not meet the threshold for diagnosis of overt diabetes is termed ‘prediabetes’ and comprises impaired fasting glucose (fasting plasma glucose 100–125 mg/dl) and impaired glucose tolerance (2-h plasma glucose 140–199 mg/dl) (19). Prediabetes, which affects 57 million adults and children in the United States (20), imparts an increased risk of both progression to overt diabetes and cardiovascular disease (21) making it a potential target for treatment.

A comparison of recommendations for treatment of prediabetes by the American Diabetes Association, American Heart Association and American College of Endocrinology is attached (Table 1). For the prevention or delay of type 2 diabetes in patients with impaired fasting glucose or impaired glucose tolerance, the American Diabetes Association suggests ‘lifestyle counselling’ [weight loss of 5–10% and at least 150 min/week of moderate activity (e.g. walking)]. In individuals at very high risk of developing diabetes (the presence of impaired fasting glucose and impaired glucose tolerance plus other risk factors), the addition of the oral antidiabetic drug metformin is recommended (19). The American Heart Association recommendations for reducing the risk of new-onset diabetes in patients with the metabolic syndrome and impaired fasting glucose or impaired glucose tolerance include weight reduction, increased physical activity, metformin, thiazolidinediones and acarbose, an intestinal α-glucosidase inhibitor (4). In a recently released consensus statement on the treatment of patients with prediabetes, the American College of Endocrinology recommends targeting hyperglycaemia and comorbid risk factors, including hypertension and dyslipidaemia, with lifestyle modifications and add-on pharmacologic therapy, where needed (22). Specifically, the guidelines, while short of clinical trial evidence, recommend that patients with prediabetes and hypertension be treated with antihypertensive agents that include those that inhibit the renin–angiotensin system (RAS) – angiotensin-converting enzyme (ACE) inhibitors and angiotensin receptor blockers (ARBs)—to the same blood pressure goal recommended for patients with overt diabetes, i.e. < 130/80 mmHg. Likewise, the consensus document recommends that lipid goals in patients with prediabetes should be the same as those with diabetes: low-density lipoprotein cholesterol < 100 mg/dl, non-high-density lipoprotein cholesterol < 130 mg/dl, and apolipoprotein B < 90 mg/dl.

**Table 1:** Recommendations for treatment of patients with pre-diabetes by the American Diabetes Association, American Heart Association and American College of Endocrinology

	American Diabetes Association (21)	American Heart Association (4)*	American College of Endocrinology (22)
Lifestyle	Weight loss of 5–10% bodyweight and ∼30 min/daymoderate-intensity physical activity	Weight loss of 7–10% body weightwithin 6–12 months ≥ 30 min/day moderate-intensity exercise Reduced intake of saturated fat(< 7% of total calories),*trans* fat, and dietary cholesterol(< 200 mg/dl); totalfat 25–35% of total calories;reduced intake of simple sugars	30–60 min moderate-intensityphysical activity/day at least 5 times/week Low-fat diet with adequate fibre
Glucose	Metformin in patients with IFG and IGTand any risk factors for diabetes†		Addition of drug therapy(e.g. acarbose, metformin) in patientswith MetS, worsening glycaemia, CVD,NA fatty liver disease, history ofgestational diabetes or PCOS
Blood pressure		BP < 140/90 mmHg BP medication(s) as needed to achieve goal BP	BP < 130/80 mmHg ACEI or ARB as first-line agent
Lipids		Depending on risk, LDL-C < 130,< 100 or < 70 mg/dl; non-HDL-C < 160,< 130 or < 100 mg/dl Lipid-lowering drug therapy with possibleaddition of fibrate or nicotinic acid	LDL-C: < 100 mg/dl Non-HDL-C: < 130 mg/dl ApoB: < 90 mg/dl
Prothrombotic state		Depending on risk, consider low-dose aspirintherapy or clopidogrel whenaspirin is contraindicated	Aspirin in therapy unless patient isat increased risk of GI, intracranialor other haemorrhagic condition

ACEI, angiotensin converting-enzyme inhibitor; ApoB, apolipoprotein B; ARB, angiotensin receptor blocker; BP, blood pressure; GI, gastrointestinal; HDL-C, high-density lipoprotein cholesterol; IFG, impaired fasting glucose; IGT, impaired glucose tolerance; LDL-C, low-density lipoprotein cholesterol; MetS, metabolic syndrome; NA, non-alcoholic; PCOS, polycystic ovary syndrome.

* American Heart Association guidelines pertain to patients with the metabolic syndrome, defined as any three of the following features: elevated waist circumference [≥ 102 cm (≥ 40 inches) in men or ≥ 88 cm (≥ 35 inches) in women], elevated triglycerides [≥ 150 mg/dl (1.7 mmol/l)] or drug treatment for elevated triglycerides, reduced HDL-C [< 40 mg/dl (< 1.03 mmol/l) in men or < 50 mg/dl (< 1.3 mmol/l)] in women or drug treatment for reduced HDL-C, elevated blood pressure (≥ 130/85 mmHg or antihypertensive drug treatment), elevated fasting glucose (≥ 100 mg/dl) or drug treatment for elevated glucose. †Risk factors for diabetes = age < 60 years, body mass index ≥ 35 kg/m^2^, family history of diabetes in first-degree relatives, elevated triglycerides, reduced HDL-C, hypertension, glycosylated haemoglobin > 6.0%.

### Lifestyle intervention

Lifestyle modification aimed at weight loss has been shown to be highly effective in preventing or delaying the development of diabetes in high-risk subjects (Table 2). In the Finnish Diabetes Prevention Study, 522 obese adults with impaired glucose tolerance were randomised to a lifestyle intervention group of individualised counselling aimed at reducing weight and increasing physical activity or to placebo (23). After a mean follow-up of 3.2 years, the incidence of diabetes was 11% in the intervention group compared with 23% in the control group, a significant 58% risk reduction (p< 0.001). Similar results were seen in the Diabetes Prevention Program (DPP), conducted in the USA, in which 3234 non-diabetic obese patients with elevated fasting and postload plasma glucose concentrations were randomised to one of the three interventions: a lifestyle programme with goals of ≥ 7% weight loss and ≥ 150 min of physical activity/week; pharmacologic therapy with metformin or placebo (24). After a mean 2.8 years, lifestyle intervention reduced the incidence of new-onset diabetes by 58% and metformin-based therapy reduced the risk of diabetes by 31%, compared with placebo (p< 0.001 for both comparisons).

**Table 2:** Effects of lifestyle modification and pharmacologic therapy with antidiabetic, anti-obesity and lipid-lowering drugs on the risk of new-onset diabetes in selected key randomised trials

Study	Comparators	Duration (years)*	Patient population	Relative risk (95% CI)	p-Value	Prespecified end-point?
**Lifestyle intervention**						
Finnish Diabetes Prevention Study (23)	Lifestyle interventionvs. placebo	3.2	522 obese patients with IGT	0.4 (0.3–0.7)	< 0.001	Yes
DPP (24)	Lifestyle interventionvs. placebo;metformin vs. placebo	2.8	3234 non-diabetic obese patientswith IFG and IGT	0.42 (0.34–0.52) 0.69 (0.57–0.83)	< 0.001 < 0.001	Yes
**Antidiabetic drugs**						
TRIPOD (26)	Troglitazone vs. placebo	2.5 (median)	266 Hispanic women withprevious gestational diabetes	0.45 (0.25–0.83)	< 0.01	Yes
STOP-NIDDM (27)	Acarbose vs. placebo	3.3	1368 patients with IGT	0.75 (0.63–0.90)	0.0015	Yes
DREAM (29)	Rosiglitazone vs. placebo	3.0 (median)	5269 patients with IFG and/or IGTbut without CVD or renal disease	0.38 (0.33–0.44)	< 0.0001	Yes
**Anti-obesity drug**						
XENDOS (31)	Orlistat (+lifestyle interventions) vs.placebo (+lifestyleinterventions)	4.0	3305 non-diabetic obese(BMI ≥ 30 kg/m^2^)patients with normal or IGT	0.63 (0.46–0.86)	0.0032	Yes
**Lipid-lowering drugs**						
WOSCOPS (32)	Pravastatin vs. placebo	4.9	5974 non-diabetic men aged45–64 yearswith dyslipidaemia and nohistory of MI	0.70 (0.50–0.99)	0.042	No
Heart ProtectionStudy (33)	Simvastatin vs. placebo	5.0	14,573 patients with occlusivearterial disease	1.15 (0.99–1.34)	ns	Yes
LIPID (34)	Pravastatin vs. placebo	6.0	6997 patients with dyslipidaemia	0.89 (0.70–1.13)	ns	No
ASCOT-LLA (35)	Atorvastatin vs. placebo	3.3 (median)	19,342 hypertensivepatients with ≥ 3other CVD risk factors	1.15 (0.91–1.44)	ns	Yes

* Mean years of follow-up unless indicated. ASCOT-LLA, Anglo-Scandinavian Cardiac Outcomes Trial-Lipid Lowering Arm; BMI, body mass index; CAD, coronary artery disease; CI, confidence interval; CVD, cardiovascular disease; DPP, Diabetes Prevention Program; DREAM, Diabetes Reduction Assessment with Ramipril and Rosiglitazone Medication; HF, heart failure; IFG, impaired fasting glucose; IGT, impaired glucose tolerance; LIPID, Long-Term Intervention with Pravastatin in Ischemic Disease; LVH, left ventricular hypertrophy; MI, myocardial infarction; ns, not significant; STOP-NIDDM, Study to Prevent Non-Insulin-Dependent Diabetes Mellitus; TRIPOD, Troglitazone in Prevention of Diabetes; WOSCOPS, West of Scotland Coronary Prevention Study; XENDOS, Xenical in the Prevention of Diabetes in Obese Subjects.

Although neither the Finnish Diabetes Prevention Study nor the DPP was designed to assess cardiovascular disease benefit, cardiovascular disease risk factors were reduced in both trials. In the Finnish study, at the end of 1 year, patients in the intervention group had significantly greater reductions in weight (p< 0.001), systolic (p= 0.007) and diastolic (p= 0.02) blood pressure, serum triglycerides (p= 0.001), and fasting plasma glucose levels (p< 0.001) (23). In the DPP, the incidence of metabolic syndrome was reduced by 41% in the lifestyle group (p< 0.001) and by 17% in the metformin group (p< 0.03) compared with placebo (25).

### Pharmacotherapy for the prevention of diabetes

A number of studies have assessed the impact of different classes of antidiabetic drugs on prevention of diabetes (Table 2) (26–28). New-onset diabetes was reduced in the Study to Prevent Non-insulin-dependent diabetes mellitus (STOP-NIDDM), in which 1368 patients with impaired glucose tolerance were treated with acarbose or placebo (27). At 39-month followup, new-onset diabetes occurred in 32% of patients in the acarbose group vs. 42% in the placebo group (risk ratio, 0.75; p= 0.0015). Treatment with acarbose was also associated with an increase in reversion to normoglycaemia. In a secondary analysis of STOP-NIDDM, acarbose also reduced the incidence of cardiovascular disease events from 4.7% to 2.1% (hazard ratio, 0.51; p= 0.03), mainly as a result of a reduction in myocardial infarction (hazard ratio, 0.09; p= 0.02) (28). Moreover, acarbose was associated with a reduced incidence of new-onset hypertension (hazard ratio, 0.66; p= 0.006), an important cardiovascular disease risk factor.

Similar results were achieved in the glycaemic arm of the Diabetes REduction Assessment with ramipril and rosiglitazone Medication (DREAM) trial, in which 5269 patients with impaired fasting glucose and/or impaired glucose tolerance and no previous cardiovascular disease or significant renal disease were randomised to treatment with rosiglitazone or placebo (and ramipril or placebo) in a 2 × 2 factorial design (29). During a median 3-year followup, 11.6% of patients in the rosiglitazone group and 26.0% in the placebo group developed the primary composite outcome of new-onset diabetes or death (hazard ratio, 0.40; p< 0.0001). When the components of the primary outcome were analysed separately, rosiglitazone was associated with a significant reduction in the incidence of new-onset diabetes (10.6% vs. 25.5%; hazard ratio, 0.38), but rates of all-cause mortality were similar in both treatment groups. Reversion to normoglycaemia was significantly more common in the rosiglitazone group (50.5% vs. 30.3%; hazard ratio, 1.71); however, there was no significant difference in cardiovascular disease event rates between the treatment groups.

The Outcome Reduction with an Initial Glargine Intervention (ORIGIN) may help to clarify the effect of glycaemic control on cardiovascular disease risk. In ORIGIN, 12,612 people aged 50 years or older with evidence of cardiovascular disease and impaired fasting glucose, impaired glucose tolerance or diabetes were randomised to treatment with insulin glargine or standard glycaemic care (and long-chain ω-3 polyunsaturated fatty acids or placebo) to determine the effect of these treatments on cardiovascular disease risk (30). The anticipated end date of the study is October 2009.

The anti-obesity drug orlistat, an inhibitor of pancreatic and gastrointestinal lipases, has been shown to reduce the risk of new-onset diabetes (Table 2). In the 4-year Xenical in the Prevention of Diabetes in Obese Subjects trial, which included 3305 non-diabetic obese (body mass index ≥ 30 kg/m^2^) individuals with normal or impaired glucose tolerance, orlistat plus lifestyle interventions reduced the incidence of new-onset diabetes by 37.3% (p= 0.0032) compared with placebo plus lifestyle intervention (31). The orlistat group also experienced significantly greater reductions in systolic and diastolic blood pressures, low-density lipoprotein cholesterol, and fasting blood glucose levels and a significantly greater increase in high-density lipoprotein cholesterol levels.

Although lipid-lowering agents such as statins have been shown to reduce the risk of cardiovascular disease in patients with diabetes, *post hoc* analyses of placebo-controlled trials have reported conflicting results regarding the effects of statins on new-onset diabetes (Table 2). In the 4.9-year West of Scotland Coronary Prevention Study, which included 5974 non-diabetic men aged 45–64 years with dyslipidaemia and no prior history of myocardial infarction, pravastatin reduced the incidence of new-onset diabetes by 30% (p= 0.042) compared with placebo (32). By contrast, in the Heart Protection Study (33), the Long-Term Intervention with Pravastatin in Ischemic Disease (34), and the Anglo-Scandinavian Cardiac Outcomes Trial-Lipid Lowering Arm (35), statin therapy had no impact on the development of diabetes.

### Role of antihypertensive agents

Patients at high risk for developing diabetes are likely to also be hypertensive. Indeed, individuals with elevated blood pressure are 2.5–5 times more likely than their normotensive counterparts to develop type 2 diabetes (36,37). Until relatively recently, discussions regarding the use of antihypertensive agents and diabetes focused on the negative metabolic effects of β-blockers and diuretics (38). More recently, however, attention has been focused on the potential metabolic benefits of RAS inhibition with ACE inhibitors and ARBs.

The effects of the β-blocker atenolol with or without a thiazide diuretic (bendroflumethiazide) were evident in the Anglo-Scandinavian Cardiac Outcomes Trial-Blood Pressure Lowering Arm (ASCOT-BPLA) (39). In the 5.5-year trial, which included 15,257 patients with hypertension and at least three other cardiovascular disease risk factors, treatment with the calcium channel blocker (CCB) amlodipine (with or without the ACE inhibitor perindopril) was associated with a 16% reduction in risk of cardiovascular disease events and a 30% reduction in risk of new-onset diabetes compared with treatment with atenolol (± bendroflumethiazide) (39). However, a new subgroup analysis of ASCOT-BPLA concluded that, along with baseline fasting plasma glucose and body mass index, the use of atenolol ± diuretic was among the major determinants of risk of new-onset diabetes (40). The authors propose that the reduction in diabetes associated with the amlodipine-based regimen may be caused by the metabolically protective effect of perindopril combined with the neutral effects of amlodipine, in contrast to the negative metabolic effects of both atenolol and bendroflumethiazide.

Analyses of several clinical trials using antihypertensive agents in patients with and without hypertension have demonstrated that RAS blockade significantly reduces the risk of new-onset diabetes in patients treated with ACE inhibitors (41–44) or ARBs (45–48), compared with diuretics, β-blockers, CCBs, or placebo (Table 3). These findings are confirmed by the results of two recent meta-analyses (49,50). In the first meta-analysis of 13 trials that included 93,451 patients without diabetes at baseline, randomisation to ACE inhibitor- or ARB-based therapy was associated with a 26% reduction in risk of developing diabetes (p < 0.001) (49). In the second, a separate network meta-analysis of 22 trials with 143,153 participants without diabetes at study outset, ARBs and ACE inhibitors were associated with the lowest proportion of subjects developing diabetes during clinical trial follow-up compared with other classes of antihypertensive agents (odds ratio, 0.57 for ARBs, p< 0.0001; and 0.67 for ACE inhibitors, p< 0.0001, using initial diuretic therapy as a standard of comparison) (50). These results are limited, however, by the fact that new-onset diabetes was not a prespecified primary or secondary outcome measure in a number of the trials included in the analysis.

**Table 3:** Effects of inhibition of the renin–angiotensin system on risk of new-onset diabetes in select key randomised controlled trials

Study	Comparators	Duration (years)*	Patient population	Relative risk (95% CI)	p-Value	Prespecified end-point?
**ACE inhibitors**						
CAPPP (41)	Captopril vs. β-blocker/diuretic	6.1	10,985 hypertensive patients	0.79 (0.67–0.94)	0.007	Yes
HOPE (42)	Ramipril vs. placebo	5	9297 patients with history of CAD, stroke,PVD, for diabetes and ≥ 1 other CVD risk factor	0.66 (0.51–0.85)	< 0.001	Yes
ALLHAT (43)	Lisinopril vs. diuretic	4.9	33,357 hypertensive patients with ≥ 1other CVD risk factor	0.70 (0.56–0.86)	< 0.001	No
PEACE (44)	Trandolapril vs. placebo	4.8 (median)	8290 with stable CAD	0.83 (0.72–0.96)	0.001	No
ASCOT-BPLA (39)	Amlodipine (±perindopril)vs. atenolol (±diuretic)	5.5 (median)	19,257 hypertensive patients with ≥ 3other CVD risk factors	0.70 (0.63–0.78)	< 0.0001	Yes
DREAM (51)	Ramipril vs. placebo	3.0 (median)	5269 patients with IFG and/or IGT but withoutCVD or renal disease	0.91 (0.80–1.03)	ns	Yes
**ARBs**						
LIFE (45)	Losartan vs. atenolol	4.8	9193 hypertensive patients with LVH	0.75 (0.63–0.86)	0.001	Yes
SCOPE (46)	Candesartan vs.placebo/other drugs	3.7	4964 hypertensive patients aged 70–89 years	0.81 (0.61–1.02)	0.09	No
CHARM (47)	Candesartan vs. placebo	3.1	7599 patients with HF	0.78 (0.64–0.96)	0.02	Yes
VALUE (48)	Valsartan vs. amlodipine	4.2	15,245 hypertensive patients withhigh risk of CVD events	0.77 (0.69–0.86)	< 0.0001	Yes
TRANSCEND (57)	Telmisartan vs. placebo	4.7 (median)	5926 patients intolerant to ACE inhibitorswith CAD, PVD, CBVD or diabeteswith end-organ damage	0.85 (0.71–1.02)	0.081	Yes
**ACE inhibitor/ARB combination**						
ONTARGET (56)	Telmisartan vs. ramipril;telmisartan + ramiprilvs. ramipril	4.7 (median)	25,620 patients with CAD, PVD, CBVDor diabetes with end-organ damage	1.12 (0.97–1.29) 0.91 (0.78–1.06)	ns ns	Yes

* Mean years of follow-up unless indicated. ACE, angiotensin-converting enzyme; ALLHAT, Antihypertensive and Lipid-Lowering treatment to Prevent Heart Attack Trial; ARB, angiotensin receptor blocker; ASCOT-BPLA, Anglo-Scandinavian Cardiac Outcomes Trial-Blood Pressure Lowering Arm; CAD, coronary artery disease; CAPP, Captopril Prevention Project; CBVD, cerebrovascular disease; CHARM, Candesartan in Heart failure – Assessment of Reduction in Morbidity and Mortality; CI, confidence interval; CVD, cardiovascular disease; DREAM, Diabetes Reduction Assessment with Ramipril and Rosiglitazone Medication; HF, heart failure; HOPE, Heart Outcomes Protection Study; IFG, impaired fasting glucose; IGT, impaired glucose tolerance; LIFE, Losartan Intervention For End-point reduction in hypertension; LVH, left ventricular hypertrophy; ns, not significant; ONTARGET, Ongoing Telmisartan Alone and in Combination with Ramipril Global Endpoint Trial; PEACE, Prevention of Events with Angiotensin-Converting Enzyme Inhibition; PVD, peripheral vascular disease; SCOPE, Study on Cognition and Prognosis in the Elderly; TRANSCEND, Telmisartan Randomised Assessment Study in ACE Intolerant Subjects with Cardiovascular Disease; VALUE, Valsartan Long-term Use Evaluation.

Several recently completed trials using ACE inhibitors or ARBs included new-onset diabetes as a prespecified outcome measure. In the hypertension arm of the 3-year DREAM trial, the incidence of the primary composite end-point of new-onset diabetes or death was similar in the ramipril and the placebo groups (18.1% vs. 19.5%; hazard ratio, 0.91; p= 0.15) (51). There was no significant difference in the incidence of new-onset diabetes or cardiovascular disease in patients treated with ramipril compared with placebo. Moreover, a new analysis of DREAM found no difference in the rates of a prespecified secondary cardiorenal end-point in either treatment group (52). The failure of ramipril to reduce the incidence of new-onset diabetes and cardiovascular disease in this trial may be explained by several features of the study design, including the trial’s short duration (median 3 years vs. median ∼4.5 years in previous RAS blocker trials) and the relatively low-risk profile of trial participants (mean age 55 years, mean blood pressure 136/83 mmHg; no history of significant cardiovascular disease or renal disease), suggesting that the degree of baseline RAS activation in the study participants was lower than that in the previous trials. Moreover, DREAM was placebo controlled, which reduced the possibility that the use of diabetogenic β-blockers or diuretics as comparator drugs confounded the results (53).

New-onset diabetes was a prespecified secondary end-point in the Valsartan Long-Term Use Evaluation (VALUE), which randomised 15,245 patients aged 50 years or older with hypertension and high risk of cardiovascular disease events to a mean of 4.2 years of valsartan- or amlodipine-based therapy (48). Although there were no statistically significant differences in the incidence of the primary composite cardiac end-point or in all-cause mortality, the incidence of new-onset diabetes was 23% lower in the valsartan group compared with the amlodipine group (13% vs. 41%; hazard ratio, 0.77). A *post hoc* analysis of the VALUE trial indicated that the risk of cardiac morbidity (defined as myocardial infarction or heart failure) in patients who developed diabetes during the 4.2-year followup was intermediate between that of those who had diabetes at baseline and those who did not develop diabetes at any point (54). Diabetes at baseline was associated with a twofold increase in risk of cardiac morbidity (hazard ratio, 2.20; p< 0.0001), while new-onset diabetes was associated with a 43% increase in risk of cardiac morbidity (p= 0.0008), primarily due to increased risk of congestive heart failure. Nonetheless, there was no difference in risk of the primary composite end-point of cardiac morbidity and mortality between those who developed new-onset diabetes and those who did not (hazard ratio, 1.10; p= 0.3447). Interestingly, new-onset diabetes was also associated with a decreased risk of all-cause mortality (hazard ratio, 0.61; p= 0.0001), possibly due to the increased use of aspirin, β-blockers, diuretics and statins in patients who developed diabetes compared with those who remained normoglycaemic (54,55).

New-onset diabetes was a secondary end-point in both the Ongoing Telmisartan Alone and in Combination with Ramipril Global Endpoint Trial (ONTARGET) (56) and the Telmisartan Randomised Assessment Study in ACE Intolerant Subjects with Cardiovascular Disease (TRANSCEND) (57). The similarly designed 56-month trials both included high-risk patients with coronary, peripheral, or cerebrovascular disease or diabetes with end-organ damage and shared the primary composite outcome of cardiovascular disease death, myocardial infarction, stroke or hospitalisation for heart failure. In ONTARGET, which randomised 25,620 patients to ramipril, telmisartan or a combination of the two drugs, the primary outcome occurred at similar rates in the three treatment groups. Likewise, although new-onset diabetes was least common in the combination therapy group (6.1% for the combination vs. 6.7% in the ramipril group and 7.5% in the telmisartan group), the differences were not significant (56). TRANSCEND compared telmisartan to placebo in 5926 patients intolerant to ACE inhibitors. Telmisartan had placebo-like tolerability. Despite a 4-mmHg weighted difference in systolic blood pressure in favour of telmisartan over placebo, the primary outcome occurred at similar rates in both treatment groups. Although new-onset diabetes occurred less frequently in the telmisartan group, the difference was not significant (57). Whether this occurred because of the excellent use of background therapy that minimised any incremental benefit that telmisartan might provide remains unclear.

Recent analyses of metabolic and clinical outcomes in non-diabetic patients in the Antihypertensive and Lipid-Lowering Treatment to Prevent Heart Attack Trial (ALLHAT) confirm the metabolic benefits of RAS inhibitors compared with diuretics but fail to demonstrate a commensurate reduction in cardiovascular disease events. ALLHAT, which did not include new-onset diabetes as a prespecified outcome, randomised patients aged 55 years or older with stage 1 or stage 2 hypertension and at least one additional cardiovascular disease risk factor to chlorthalidone, amlodipine or lisinopril for a mean of 4.9 years (43). Among patients without diabetes at baseline, the incidence of diabetes at 4 years was the greatest with chlorthalidone (11.6%), lower with amlodipine (9.8%; p= 0.04 vs. chlorthalidone), and the lowest in those treated with lisinopril (8.1%; p< 0.001 vs. chlorthalidone) (43). However, in patients with the metabolic syndrome at baseline, the incidence of new-onset diabetes was almost twice as high as in those without the metabolic abnormality at baseline: 17.1% in the chlorthalidone group, 16.0% in the amlodipine group and 12.6% in the lisinopril group (p< 0.05 for lisinopril vs. chlorthalidone), compared with rates of 7.7%, 4.2% and 4.7% for chlorthalidone, amlodipine and lisinopril, respectively (p< 0.05 for both amlodipine and lisinopril vs. chlorthalidone) (58). Despite these differences, the risk of combined cardiovascular disease events was similar in those with the metabolic syndrome as in those without, and in those who developed diabetes and in those who did not (58). In a separate subgroup analysis that compared cardiovascular disease outcomes by race in non-diabetic patients with and without the metabolic syndrome, long-term cardiovascular outcomes were similar across the treatment groups, with the lack of cardiovascular benefit especially striking in black patients with the metabolic syndrome (59).

These analyses have not resolved the controversy regarding the relationship between new-onset diabetes and cardiovascular disease risk and have led some investigators to question whether diuretic- or β-blocker-associated diabetes confers the same clinical implications as new-onset diabetes that develops outside the setting of antihypertensive therapy (60).

Further clarification of the comparative value of antihypertensive combinations comparing the use of a glucose neutral agent such as a CCB with a thiazide-type diuretic will come from the Avoiding Cardiovascular Events Through Combination Therapy in Patients Living With Systolic Hypertension trial. In this trial, the high-risk hypertensive patients were initially randomised to a fixed-dose combination of either an ACE inhibitor/thiazide-type diuretic or an ACE inhibitor/CCB. New onset diabetes is a prespecified secondary end-point (61).

The question of whether preventing new-onset diabetes is associated with a reduction in cardiovascular disease events may be resolved with the results of two ongoing large-scale clinical trials – Nateglinide And Valsartan in Impaired Glucose Tolerance Outcomes Research (NAVIGATOR) (62) and ACE Inhibitor-based vs. Diuretic-based Antihypertensive Primary Treatment in Patients with PreDiabetes (ADaPT) (63). NAVIGATOR is a multinational, double-blind, placebo-controlled, 2 × 2 factorial study to determine the effects of the meglitinide antidiabetic drug nateglinide and of valsartan on two primary outcomes: prevention of new-onset diabetes and prevention of cardiovascular disease events (death, myocardial infarction, stroke and hospitalisation for heart failure). A total of 9306 participants aged 50 years or older with impaired fasting glucose and known cardiovascular disease or aged 55 years or older with impaired glucose tolerance and 1 or more cardiovascular disease risk factors were randomised in a 1 : 1 : 1 : 1 ratio to one of four possible treatment combinations: nateglinide with valsartan, nateglinide with valsartan-placebo, nateglinide-placebo with valsartan or nateglinide-placebo with valsartan-placebo. The results of this events-driven trial are expected to be reported in 2009 (62). ADaPT, which includes 2015 patients with hypertension, impaired fasting glucose, and A1C 6–6.5%, is an open-label trial designed to compare the effect on new-onset diabetes of antihypertensive treatment based on ramipril vs. treatment based on diuretics or β-blockers. The results of the 4-year study are expected in 2010 (63).

In the interim, the effects of antihypertensive therapy and the presence of comorbid risk factors on the risk of new-onset diabetes and cardiovascular disease should be carefully evaluated for each patient. Indeed, in *post hoc* analyses of ASCOT-BPLA (40) and VALUE (64), baseline fasting plasma glucose level and body mass index – two measurable, modifiable risk factors – were the strongest predictors of new-onset diabetes. In ASCOT-BPLA, randomisation of the amlodipine ± perindopril treatment arm was the strongest protective factor (40), highlighting the importance of including a RAS inhibitor as part of the antihypertensive regimen in patients at high risk for diabetes and cardiovascular disease.

### Possible mechanisms

The mechanisms by which RAS inhibition reduces the development of diabetes remain to be established. ACE inhibitors and ARBs improve insulin sensitivity, possibly caused by their vasodilatory effects, which result in increased blood flow and increased insulin delivery to peripheral skeletal muscles (65–67). ACE inhibitors and ARBs may also improve glucose metabolism by enhancing insulin receptor signalling in skeletal muscle and fat cells (68). RAS blockade is associated with a reduction in renal potassium loss, which may lead to enhanced β-cell secretion of insulin (66,68) and may protect pancreatic islets from glucotoxicity and oxidative stress (67). In addition, ARBs increase levels of adiponectin, an adipose-derived protein thought to enhance insulin sensitivity (67,69). Finally, some ARBs activate peroxisome proliferator-activated receptor-γ, a well-established target for insulin-sensitising antidiabetic drugs (66,69).

## Conclusions

Diabetes is a worldwide epidemic with the substantial health ramifications. Because cardiovascular disease is the leading cause of death in patients with diabetes, the prevention of diabetes has the potential to reduce the burden of cardiovascular disease. Evidence suggests that lifestyle modifications aimed at weight reduction and increased physical activity and antidiabetic pharmacologic interventions reduce the risk of new-onset diabetes. Although there is ample evidence that antihypertensive therapy with RAS inhibitors is associated with a reduced risk of new-onset diabetes compared with other classes of antihypertensive drugs, the prognostic significance of this differential effect remains controversial. No study using antihypertensive therapy has yet demonstrated a commensurate reduction in cardiovascular disease risk, and secondary analyses of trials of RAS blockers have provided conflicting results. It appears reasonable to avoid traditional β-blocker and thiazide-type diuretic therapy in the minority of patients who have impaired fasting glucose or impaired glucose tolerance and whose blood pressure can be controlled on single-agent antihypertensive therapy. In these patients, the use of ACE inhibitor and ARB therapy, like in those with the metabolic syndrome, may have an advantage in decreasing the subsequent risk for new-onset diabetes. When blood pressure is not effectively controlled with ACE inhibitor or ARB therapy, the addition of a thiazide-type diuretic is clearly indicated as the benefits of achieving blood pressure control appear to outweigh any negative effects on glucose metabolism. Whether a CCB should be added before a thiazide-type diuretic remains uncertain. The results of the large-scale NAVIGATOR study may help resolve the issue of whether treatment of high-risk patients with an antidiabetic drug or an ARB reduces the incidence of new-onset diabetes and cardiovascular disease.

For now, lifestyle measures including weight loss and exercise as well as the importance of addressing global cardiovascular disease risk through the use of statin and aspirin therapy may be a more effective strategy to improve vascular health and limit insulin resistance in patients with hypertension. Additionally, achieving blood pressure control with agents that do not worsen insulin resistance should be attempted; failing that, vasodilating β-blockers and low-dose diuretics must be used to reduce risk. Physicians should be aware that in most cases multiple antihypertensive agents will be needed. While keeping serum potassium levels normal, diuretics should be considered when blood pressure goals have not been achieved in patients with impaired fasting glucose. When used in this setting, although new-onset diabetes may be more likely to occur, cardiovascular outcomes are still improved by achieving additional blood pressure reduction.
